# Service Quality and Residents’ Preferences for Facilitated Self-Service Fundus Disease Screening: Cross-Sectional Study

**DOI:** 10.2196/45545

**Published:** 2024-04-17

**Authors:** Senlin Lin, Yingyan Ma, Yanwei Jiang, Wenwen Li, Yajun Peng, Tao Yu, Yi Xu, Jianfeng Zhu, Lina Lu, Haidong Zou

**Affiliations:** 1 Shanghai Eye Diseases Prevention &Treatment Center/ Shanghai Eye Hospital School of Medicine Tongji University Shanghai China; 2 National Clinical Research Center for Eye Diseases Shanghai China; 3 Shanghai Engineering Research Center of Precise Diagnosis and Treatment of Eye Diseases Shanghai China; 4 Shanghai General Hospital School of Medicine Shanghai Jiao Tong University Shanghai China; 5 Shanghai Hongkou Center for Disease Control and Prevention Shanghai China; 6 School of Management Fudan University Shanghai China

**Keywords:** digital technology, screening, self-service, eye disease, health economics evaluation, health technology assessment, disease screening, artificial intelligence, AI, eye, community, effectiveness, screening efficiency, safety

## Abstract

**Background:**

Fundus photography is the most important examination in eye disease screening. A facilitated self-service eye screening pattern based on the fully automatic fundus camera was developed in 2022 in Shanghai, China; it may help solve the problem of insufficient human resources in primary health care institutions. However, the service quality and residents’ preference for this new pattern are unclear.

**Objective:**

This study aimed to compare the service quality and residents’ preferences between facilitated self-service eye screening and traditional manual screening and to explore the relationships between the screening service’s quality and residents’ preferences.

**Methods:**

We conducted a cross-sectional study in Shanghai, China. Residents who underwent facilitated self-service fundus disease screening at one of the screening sites were assigned to the exposure group; those who were screened with a traditional fundus camera operated by an optometrist at an adjacent site comprised the control group. The primary outcome was the screening service quality, including effectiveness (image quality and screening efficiency), physiological discomfort, safety, convenience, and trustworthiness. The secondary outcome was the participants’ preferences. Differences in service quality and the participants’ preferences between the 2 groups were compared using chi-square tests separately. Subgroup analyses for exploring the relationships between the screening service’s quality and residents’ preference were conducted using generalized logit models.

**Results:**

A total of 358 residents enrolled; among them, 176 (49.16%) were included in the exposure group and the remaining 182 (50.84%) in the control group. Residents’ basic characteristics were balanced between the 2 groups. There was no significant difference in service quality between the 2 groups (image quality pass rate: *P*=.79; average screening time: *P*=.57; no physiological discomfort rate: *P*=.92; safety rate: *P*=.78; convenience rate: *P*=.95; trustworthiness rate: *P*=.20). However, the proportion of participants who were willing to use the same technology for their next screening was significantly lower in the exposure group than in the control group (*P*<.001). Subgroup analyses suggest that distrust in the facilitated self-service eye screening might increase the probability of refusal to undergo screening (*P*=.02).

**Conclusions:**

This study confirms that the facilitated self-service fundus disease screening pattern could achieve good service quality. However, it was difficult to reverse residents’ preferences for manual screening in a short period, especially when the original manual service was already excellent. Therefore, the digital transformation of health care must be cautious. We suggest that attention be paid to the residents’ individual needs. More efficient man-machine collaboration and personalized health management solutions based on large language models are both needed.

## Introduction

Vision impairment and blindness are caused by a variety of eye diseases, including cataracts, glaucoma, uncorrected refractive error, age-related macular degeneration, diabetic retinopathy, and other eye diseases [1]. They not only reduce economic productivity but also harm the quality of life and increase mortality [2-6]. In 2020, an estimated 43.3 million individuals were blind, and 1.06 billion individuals aged 50 years and older had distance or near vision impairment [7]. With an increase in the aging population, the number of individuals affected by vision loss has increased substantially [1].

High-quality public health care for eye disease prevention, such as effective screening, can assist in eliminating approximately 57% of all blindness cases [8]. Digital technologies, such as telemedicine, 5G telecommunications, the Internet of Things, and artificial intelligence (AI), have provided the potential to improve the accessibility, availability, and productivity of existing resources and the overall efficiency of eye care services [9,10]. The use of digital technology not only reduces the cost of eye disease screening and improves its efficiency, but also assists residents living in remote areas to gain access to eye disease screening [11-13]. Therefore, an increasing number of countries (or regions) are attempting to establish eye screening systems based on digital technology [9].

Fundus photography is the most important examination in eye disease screening because the vast majority of diagnoses of blinding retinal diseases are based on fundus photographs. Diagnoses can be made by human experts or AI software. However, traditional fundus cameras must be operated by optometrists, who are usually in short supply in primary health care institutions when faced with the large demand for screening services.

Fortunately, the fully automatic fundus camera has been developed on the basis of digital technologies including AI, industrial automation, sensors, and voice navigation. It can automatically identify the person’s left and right eyes, search for pupils, adjust the lens position and shooting focus, and provide real-time voice feedback during the process, helping the residents to understand the current inspection steps clearly and cooperatively complete the inspection. Therefore, a facilitated self-service eye screening pattern has been newly established in 2022 in Shanghai, China.

However, evidence is inadequate about whether this new screening pattern performs well and whether the residents prefer it. Therefore, this cross-sectional study aims to compare the service quality and residents’ preferences of this new screening pattern with that of the traditional screening pattern. We aimed to (1) investigate whether the facilitated self-service eye screening can achieve service quality similar to that of traditional manual screening, (2) compare residents’ preferences between the facilitated self-service eye screening and traditional manual screening, and (3) explore the relationship between the screening service quality and residents’ preferences.

## Methods

### Study Setting

**Figure 1:**
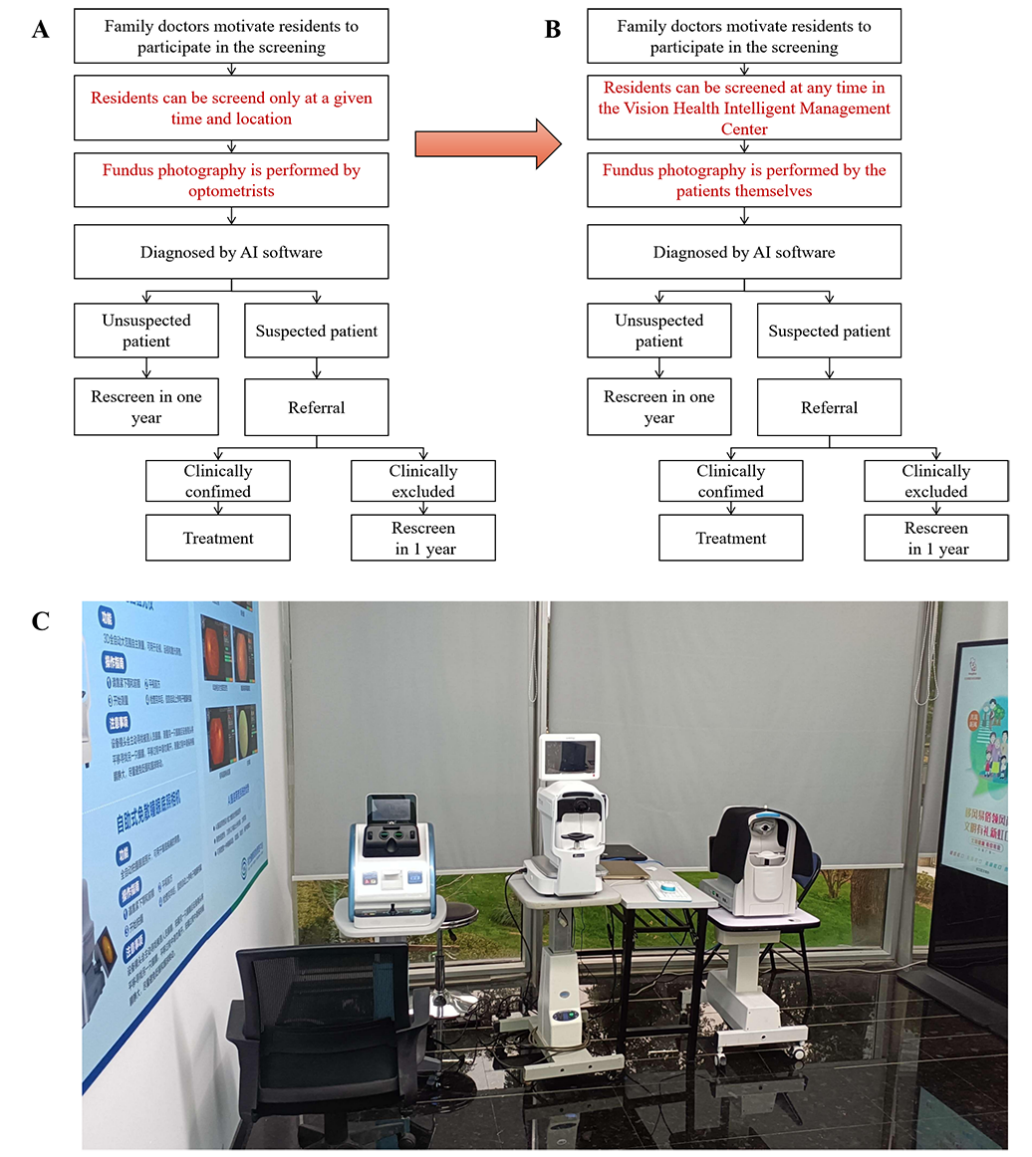
Comparison of the facilitated self-service fundus disease screening pattern and the traditional telemedicine screening pattern. (A) Workflow of the traditional telemedicine screening pattern. (B) Workflow of the facilitated self-service eye screening pattern. (C) Actual image of a Vision Health Intelligent Management Center. It is a site built in the communities for residents to receive eye disease screening and health management, with the facilitated self-service eye screening pattern. Three fully automatic self-service ophthalmic examination devices have been equipped. The device on the left in the photo is a fully automatic self-service visual acuity, the one in the middle is a fully automatic self-service computer optometry device, and the one on the right one is a fully automatic self-service nonmydriatic fundus camera. AI: artificial intelligence.

This study was conducted in Shanghai, China, in 2022. Since 2010, Shanghai has conducted an active community-based fundus disease telemedicine screening program. After 2018, an AI model was adopted (Figure 1). At the end of 2021, the fully automatic fundus camera was adopted, and the facilitated self-service fundus disease screening pattern was established (Figure 1). Within this new pattern, residents could perform fundus photography by themselves without professionals’ assistance (Multimedia Appendix 1). The fundus images were sent to the cloud server center of the AI model, and the screening results were fed back immediately.

### Study Design

We conducted a cross-sectional study at 2 adjacent screening sites. These 2 sites were expected to be very similar in terms of their socioeconomic and educational aspects since they were located next to each other. One site provided facilitated self-service fundus disease screening, and the residents who participated therein comprised the exposure group; the other site provided screening with a traditional fundus camera operated by an optometrist, and the residents who participated therein comprised the control group. All the adult residents could participant in our screening program, but their data were used for analysis only if they signed the informed consent form. Residents could opt out of the study at any time during the screening.

**Figure 2:**
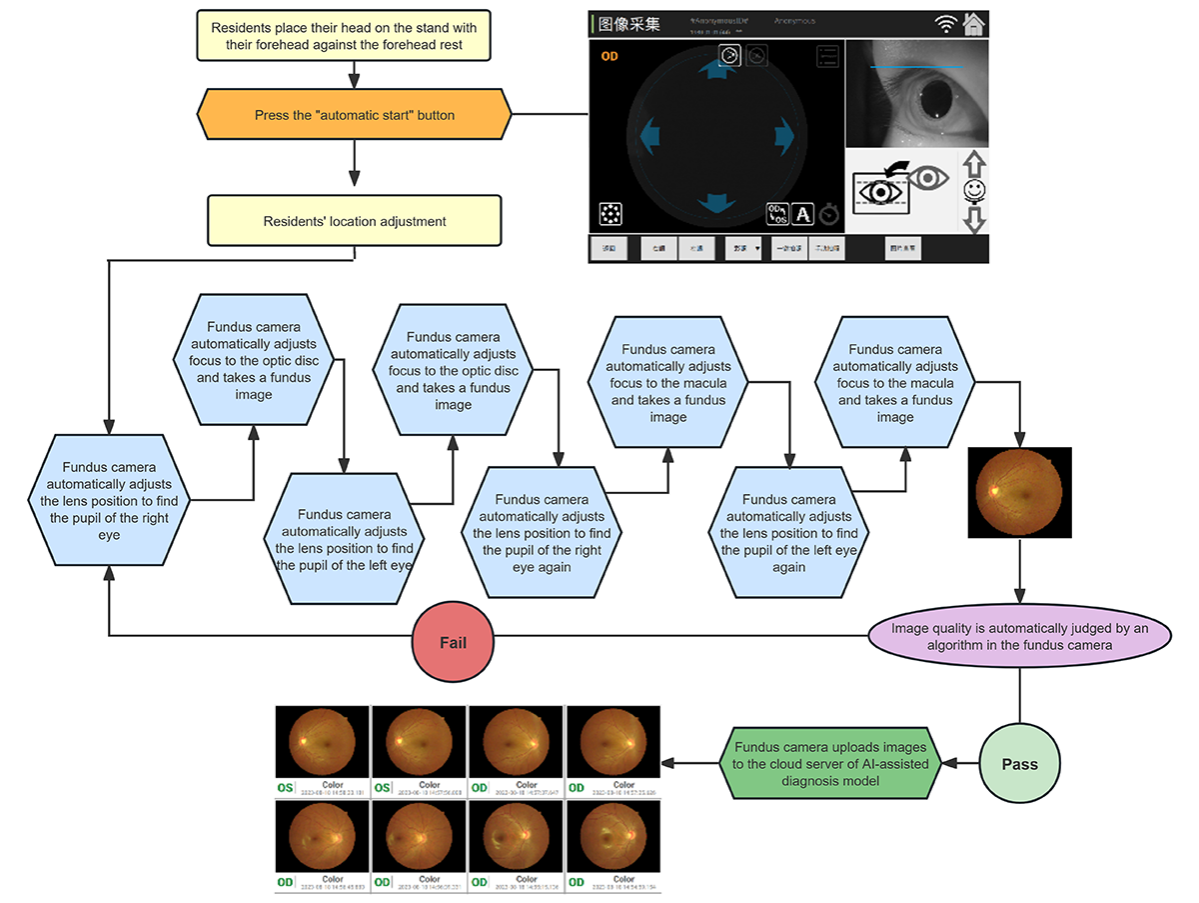
Workflow of the fully automatic self-service nonmydriatic fundus camera. AI: artificial intelligence.

In the exposure group, the residents were assessed using an updated version of the nonmydriatic fundus camera Kestrel 3100m (Shanghai Top View Industrial Co Ltd) with a self-service module. In the process of fundus photography, the residents pressed the “Start” button by themselves. All checking steps (including focusing, shooting, and image quality review) were undertaken automatically by the fundus camera (Figure 2). Screening data were transmitted to the AI algorithm on a cloud-based server center through the telemedicine platform, and the screening results were fed back immediately. Residents were fully informed that the assessment was fully automated and not performed by the optometrist.

In the control group, the residents were assessed using the basic version of the same nonmydriatic fundus camera. The optical components were identical to those in the exposure group but without the self-service module. In the process of fundus photography, all steps were carried out by the optometrist (including focusing, shooting, and image quality review). Screening data were transmitted to the AI algorithm on a cloud-based server center through the telemedicine platform, and the screening results were fed back immediately. Residents were also fully informed.

### Measures and Outcomes

The primary outcome was the screening service’s quality. Based on the World Health Organization’s recommendations for the evaluation of AI-based medical devices [14] and the European Union’s Assessment List for Trustworthy Artificial Intelligence [15], 5 dimensions were selected to reflect the service quality of eye disease screening: effectiveness, physiological discomfort, safety, convenience, and trustworthiness.

Furthermore, effectiveness was based on 2 indicators: image quality and screening efficiency. A staff member recorded the time required for each resident to take fundus photographs (excluding the time taken for diagnosis) at the screening site. Then, a professional ophthalmologist evaluated the quality of each fundus photograph after the on-site experiment. The ophthalmologist was blinded to the grouping of participants. Image quality was assessed on the basis of the image quality pass rate, expressed as the number of eyes with high-quality fundus images per 100 eyes. Screening efficiency was assessed on the basis of the average screening time, expressed as the mean of the time required for each resident to take fundus photographs.

To assess physiological discomfort, safety, convenience, and trustworthiness of screening services, residents were asked to finish a questionnaire just after they received the screening results. A 5-point Likert scale was adopted for each dimension, from the best to the worst, except for the physiological discomfort (Multimedia Appendix 2). A no physiological discomfort rate was expressed as the number of residents who chose the “There is no physiological discomfort during the screening” per 100 individuals in each group. Safety rate is expressed as the number of residents who chose “The screening is very safe” or “The screening is safe” per 100 individuals in each group. Convenience rate is expressed as the number of residents who chose “The screening is very convenient” or “The screening is convenient” per 100 individuals in each group. The trustworthiness rate is expressed as the number of residents who chose “The screening result is very trustworthy” or “The screening result is trustworthy” per 100 individuals in each group.

The secondary outcome was the preference rate, expressed as the number of residents who were willing to use the same technology for their next screening per 100 individuals. In detail, in the exposure group, the preference rate was expressed as the number of the residents who preferred facilitated self-service eye screening per 100 individuals, while in the control group, it was expressed as the number of residents who preferred traditional manual screening per 100 individuals.

To understand the residents’ preference, a video displaying the processes of both facilitated self-service eye screening and traditional manual screening was shown to the residents. Then, the following question was asked: “At your next eye disease screening, you can choose either facilitated self-service eye screening or traditional manual screening. Which one do you prefer?” A total of 4 alternatives were set: “Prefer traditional manual screening,” “Prefer facilitated self-service eye screening,” “Both are acceptable,” and “Neither is acceptable (Refusal of screening).” Each resident could choose only 1 option, which best reflected their preference.

### Sample Size

The rule of events per variable was used for sample size estimation. In this study, 2 logit models were established for the 2 groups separately, each containing 8 independent variables. We set 10 events per variable in general. According to a previous study [16], when the decision-making process had high uncertainty, the proportion of individuals who preferred the algorithms was about 50%. This led us to arrive at a sample size of 160 (8 variables multiplied by 10 events each, with 50% of individuals potentially preferring facilitated screening [ie, 50% of 8×10]) for each group.

### Analysis

Every dimension of the screening service quality and the preference rate were calculated separately. Chi-square and *t* tests were used to test whether the service quality or the residents’ preferences differed between the 2 groups. A total of 7 hypotheses were tested, as shown in Textbox 1.

**Study hypotheses tested.**

1. H1: image quality pass rate _exposure group_≠ image quality pass rate _control group_
  H0: image quality pass rate _exposure group_=image quality pass rate _control group_
2. H1: screening time _exposure group_≠screening time _control group_
  H0: screening time _exposure group_=screening time _control group_
3. H1: no discomfort rate_exposure group_≠no discomfort rate _control group_
  H0: no discomfort rate _exposure group_ = no discomfort rate _control group_
4. H1: safety rate_exposure group_≠safety rate _control group_
  H0: safety rate _exposure group_ = safety rate _control group_
5. H1: convenience rate_exposure group_≠convenience rate _control group_
  H0: convenience rate _exposure group_ = convenience rate _control group_
6. H1: trustworthiness rate _exposure group_≠trustworthiness rate _control group_
  H0: trustworthiness rate _exposure group_ = trustworthiness rate _control group_
7. H1: preference rate _exposure group_≠preference rate _control group_
  H0: preference rate _exposure group_ = preference rate _control group_

If any of the hypotheses among hypotheses 1-6 (Textbox 1) were significant, it indicated that the service quality was different between facilitated self-service eye screening and traditional manual screening. If hypothesis 7 was significant, it meant that the residents’ preference for facilitated self-service eye screening was different from that for traditional manual screening.

Additionally, subgroup analyses in the exposure and control groups were conducted to explore the relationships between the screening service quality and the residents’ preferences, using generalized logit models. The option “Prefer facilitated self-service eye screening” was used as the reference level for the dependent variable in the models. The independent variables included age, sex, image quality, screening efficiency, physiological discomfort, safety, convenience, and trustworthiness. All statistics were performed using SAS (version 9.4; SAS Institute).

### Ethical Considerations

The study adhered to the ethical principles of the Declaration of Helsinki and was approved by the Shanghai General Hospital Ethics Committee (2022SQ272). All participants provided written informed consent before participating in this study. The study data were anonymous, and no identification of individual participants in any images of the manuscript or supplementary material is possible.

## Results

### Participants’ Characteristics

A total of 358 residents enrolled; among them, 176 (49.16%) were in the exposure group and the remaining 182 (50.84%) were in the control group. Residents’ basic characteristics were balanced between the 2 groups. The mean age was 65.05 (SD 12.28) years for the exposure group and 63.96 (SD 13.06) years for the control group; however, this difference was nonsignificant (*P*=.42). The proportion of women was 67.05% (n=118) for the exposure group and 62.09% (n=113) for the control group; this difference was also nonsignificant between the 2 groups (*P*=.33).

### Screening Service Quality

**Figure 3:**
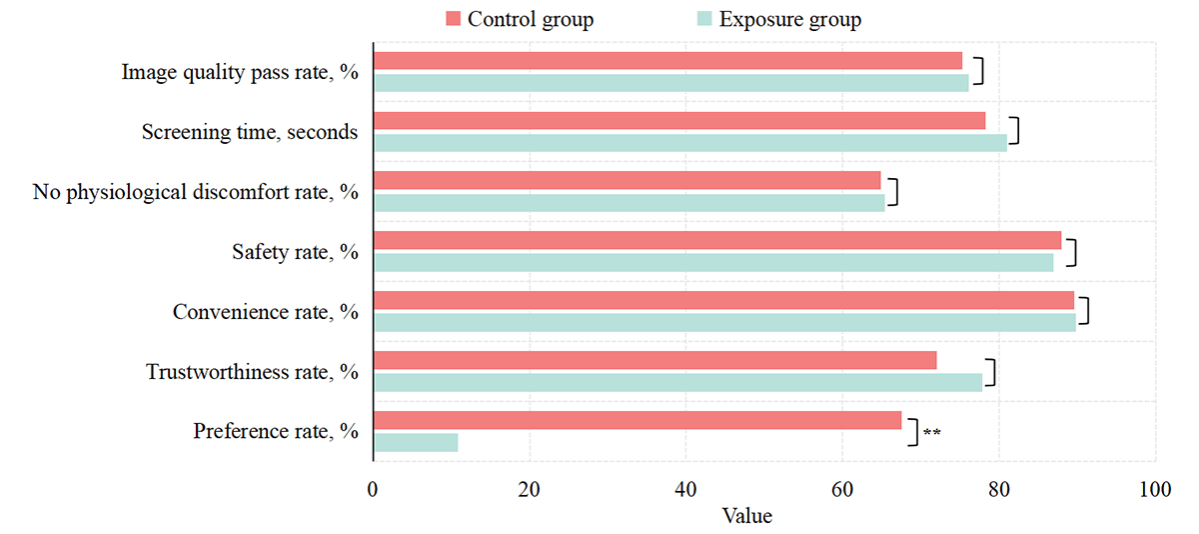
Screening effectiveness and residents’ preferences in the exposure and control groups. There was no significant difference between the 2 groups in the measures reflecting service quality. However, the proportion of participants who were willing to use the same technology for their next screening in the exposure group was much lower than in the control group (*P*<.001).

In the exposure group, high-quality fundus images were obtained for 268 out of 352 eyes (image quality pass rate=76.14%; Figure 3). The average screening time was 81.03 (SD 36.98) seconds (Figure 3). In the control group, high-quality fundus images were obtained for 274 out of 364 eyes (image quality pass rate=75.27%; Figure 3). The average screening time was 78.22 (SD 54.01) seconds (Figure 3). There was no significant difference in the image quality pass rate (*χ*^2^_1_=0.07, *P*=.79) and average screening time (*t_321.01_*=–0.58 [Welch–Satterthwaite–adjusted *df*], *P*=.56) between the 2 groups (Figure 3).

For the other dimensions, detailed information is shown in Figure 3. There were no significant differences between any of these rates between the 2 groups (no physiological discomfort rate: *χ*^2^_1_=0.01, *P*=.92; safety rate: *χ*^2^_1_=0.08, *P*=.78; convenience rate: *χ*^2^_1_=0.004, *P*=.95; trustworthiness rate: *χ*^2^_1_=1.63, *P*=.20).

### Residents’ Preferences

In the exposure group, 120 (68.18%) residents preferred traditional manual screening, 19 (10.80%) preferred facilitated self-service eye screening, 19 (10.80%) preferred both, and the remaining 18 (10.23%) preferred neither. In the control group, 123 (67.58%) residents preferred traditional manual screening, 14 (7.69%) preferred facilitated self-service eye screening, 20 (10.99%) preferred both, and the remaining 25 (13.74%) preferred neither.

The proportion of residents who chose the category “Prefer facilitated self-service eye screening” in the exposure group was significantly lower than that of residents who chose the category “Prefer traditional manual screening” in the control group (*χ*^2^_1_=120.57, *P*<.001; Figure 3).

### Subgroup Analyses

In the exposure group, 4 generalized logit models were generated (Table 1). Regarding the effectiveness of facilitated self-service eye screening, neither the image quality nor the screening time had an impact on the residents’ preferences. Regarding the other dimensions for facilitated self-service eye screening service quality, models 3 and 4 demonstrated that distrust in the results of facilitated self-service eye screening might decrease the probability of preferring this screening service and increase the probability of preferring neither of the 2 screening services.

**Table 1:** Subgroup analysis for exploring the factors affecting residents’ preferences^a^ in the exposure group (n=176)^b^ (models 1-4).

		Effectiveness scores, mean (SE; *P* value)			
		Model 1	Model 2	Model 3	Model 4
**Prefer traditional manual screening**					
	Male gender	0.35 (0.30; .24)	0.36 (0.30; .23)	0.37 (0.31; .24)	0.37 (0.31; .24)
	Age	0.02 (0.02; .34)	0.02 (0.02; .28)	0.02 (0.02; .32)	0.03 (0.02; .23)
	Number of high-quality images	—^c^	0.05 (0.37; .89)	—	0.12 (0.37; .75)
	Screening time	—	–0.01 (0.01; .43)	—	–0.01 (0.01; .37)
	Feel that the facilitated self-service eye screening is uncomfortable	—	—	0.1 (0.27; .71)	0.1 (0.28; .73)
	Feel that the facilitated self-service eye screening is unsafe	—	—	6.37 (105.60; .95)	6.78 (147.30; .96)
	Feel that the facilitated self-service eye screening is inconvenient	—	—	–1.23 (0.75; .10)	–1.29 (0.75; .09)
	Distrust the results of the facilitated self-service eye screening	—	—	–0.18 (0.38; .64)	–0.20 (0.38; .59)
**Prefer both traditional and facilitated self-service eye screening**					
	Male gender	0.53 (0.37; .15)	0.54 (0.37; .14)	0.62 (0.39; .11)	0.65 (0.39; .10)
	Age	0.03 (0.03; .32)	0.02 (0.03; .36)	0.03 (0.03; .35)	0.03 (0.03; .37)
	Number of high-quality images	—	0.08 (0.48; .87)	—	0.21 (0.51; .68)
	Screening time	—	0 (0.01; .66)	—	0 (0.01; .55)
	Feel that the facilitated self-service eye screening is uncomfortable	—	—	–0.04 (0.37; .90)	–0.02 (0.38; .96)
	Feel that the facilitated self-service eye screening is unsafe	—	—	6.08 (105.60; .95)	6.47 (147.30; .96)
	Feel that the facilitated self-service eye screening is inconvenient	—	—	0.66 (0.76; .38)	0.70 (0.78; .37)
	Distrust the results of the facilitated self-service eye screening	—	—	–1.57 (0.83; .06)	–1.67 (0.86; .05)
**Prefer neither traditional nor facilitated self-service screening (refusal for screening)**					
	Male gender	0.21 (0.39; .58)	0.22 (0.39; .58)	–0.07 (0.46; .87)	–0.10 (0.47; .84)
	Age	0.03 (0.03; .26)	0.04 (0.03; .22)	0.02 (0.03; .48)	0.03 (0.04; .45)
	Number of high-quality images	—	0.20 (0.49; .68)	—	0.21 (0.62; .74)
	Screening time	—	0 (0.01; .81)	—	0 (0.01; .99)
	Feel that the facilitated self-service eye screening is uncomfortable	—	—	0.03 (0.46; .95)	0.01 (0.47; .98)
	Feel that the facilitated self-service eye screening is unsafe	—	—	6.38 (105.60; .95)	6.77 (147.30; .96)
	Feel that the facilitated self-service eye screening is inconvenient	—	—	0 (0.74; >.99)	–0.06 (0.75; .94)
	Distrust the results of the facilitated self-service eye screening	—	—	1.32 (0.55; .02)	1.31 (0.55; .02)

^a^Age and gender were adjusted in model 1. Age, gender, image quality, and screening efficiency were adjusted in model 2. Age, gender, physiological discomfort, safety, convenience, and trustworthiness were adjusted in model 3. Age, gender, image quality, screening efficiency, physiological discomfort, safety, convenience, and trustworthiness were adjusted in model 4.

^b^In the exposure group, distrust in the results of facilitated self-service eye screening might decrease the probability of preferring this screening service and increase the probability of preferring neither the traditional nor the facilitated self-service screening services.

^c^Not available.

In the control group, another 4 generalized logit models were generated (Table 2). Men were more likely to choose a preference both screening services. The probability of preferring manual screening might increase with age, as long as the probability of preferring facilitated self-service eye screening decreased. Regarding the effectiveness of traditional manual screening, neither the image quality pass rate nor the screening time had an impact on the residents’ preferences. For the other dimensions of the quality of traditional manual screening, models 7 and 8 showed that if the residents feel unsafe about traditional manual screening, their preference for traditional manual screening might decrease, and they might turn to facilitated self-service eye screening.

**Table 2:** Subgroup analysis for exploring the factors affecting residents’ preferences^a^ in the control group (n=182)^b^ (models 5-8).

		Effectiveness scores, mean (SE; *P* value)			
		Model 5	Model 6	Model 7	Model 8
**Prefer traditional manual screening**					
	Male gender	0.49 (0.36; .17)	0.51 (0.36; .16)	0.42 (0.37; .27)	0.43 (0.38; .26)
	Age	0.07 (0.02; .002)	0.07 (0.02; .002)	0.06 (0.02; .006)	0.07 (0.02; .004)
	Number of high-quality images	—^c^	0.19 (0.47; .69)	—	0.26 (0.52; .61)
	Screening time	—	0 (0.01; .93)	—	0 (0.01; .86)
	Feel that the facilitated self-service eye screening is uncomfortable	—	—	0.40 (0.40; .32)	0.38 (0.40; .34)
	Feel that the facilitated self-service eye screening is unsafe	—	—	–1.36 (0.51; .007)	–1.45 (0.53; .006)
	Feel that the facilitated self-service eye screening is inconvenient	—	—	6.33 (151.00; .97)	6.37 (130.50; .96)
	Distrust the results of the facilitated self-service eye screening	—	—	–0.24 (0.35; .48)	–0.27 (0.35; .44)
**Prefer both traditional and facilitated self-service eye screening**					
	Male gender	0.94 (0.41; .02)	0.92 (0.42; .03)	0.94 (0.44; .03)	0.96 (0.45; .03)
	Age	0.03 (0.02; .22)	0.03 (0.03; .27)	0.02 (0.03; .55)	0.02 (0.03; .58)
	Number of high-quality images	—	–0.27 (0.54; .62)	—	–0.16 (0.60; .79)
	Screening time	—	0 (0.01; .85)	—	0 (0.01; .84)
	Feel that the facilitated self-service eye screening is uncomfortable	—	—	0.52 (0.46; .26)	0.61 (0.46; .19)
	Feel that the facilitated self-service eye screening is unsafe	—	—	–8.03 (126.40; .95)	–8.84 (79.57; .91)
	Feel that the facilitated self-service eye screening is inconvenient	—	—	8.63 (151.00; .95)	9.64 (130.50; .91)
	Distrust the results of the facilitated self-service eye screening	—	—	–0.56 (0.52; .29)	–0.46 (0.51; .37)
**Prefer neither traditional nor facilitated self-service screening (refusal for screening)**					
	Male gender	0.48 (0.39; .23)	0.38 (0.41; .36)	0.64 (0.47; .17)	0.46 (0.49; .35)
	Age	0.01 (0.02; .72)	0.01 (0.02; .58)	–0.01 (0.03; .80)	0 (0.03; .89)
	Number of high-quality images	—	1.21 (0.67; .07)	—	1.07 (0.80; .18)
	Screening time	—	0.02 (0.01; .06)	—	0.02 (0.01; .19)
	Feel that the facilitated self-service eye screening is uncomfortable	—	—	0.71 (0.51; .16)	0.54 (0.52; .30)
	Feel that the facilitated self-service eye screening is unsafe	—	—	–1.32 (0.80; .10)	–0.90 (0.79; .25)
	Feel that the facilitated self-service eye screening is inconvenient	—	—	8.21 (151.00; .96)	7.55 (130.50; .95)
	Distrust the results of the facilitated self-service eye screening	—	—	0.79 (0.46; .09)	0.62 (0.47; .19)

^a^Age and gender were adjusted in model 5. Age, gender, image quality, and screening efficiency were adjusted in model 6. Age, gender, physiological discomfort, safety, convenience, and trustworthiness were adjusted in model 7. Age, gender, image quality, screening efficiency, physiological discomfort, safety, convenience, and trustworthiness were adjusted in model 8.

^b^In the control group, if the residents feel unsafe about traditional manual screening, their preference for traditional manual screening might decrease, and they might turn to facilitated self-service eye screening.

^c^Not available.

## Discussion

A new fundus disease screening pattern was established using the fully automatic fundus camera without any manual intervention. Our findings suggest that facilitated self-service eye screening can achieve a service quality similar to that of traditional manual screening. The study further evaluated the residents’ preferences and associated factors for the newly established self-service fundus disease screening. Our study found that the residents’ preference for facilitated self-service eye screening is significantly less than that for traditional manual screening. This implies that the association between the service quality of the screening technology and residents’ preferences was weak, suggesting that aversion to the algorithm might exist. In addition, the subgroup analyses suggest that even the high quality of facilitated self-service eye screening cannot increase the residents’ preference for this new screening pattern. Worse still, distrust in the results of this new pattern may lead to lower usage of eye disease screening services as a whole. To the best of our knowledge, this study is one of the first to evaluate service quality and residents’ preferences for facilitated self-service fundus disease screening.

Previous studies have suggested that people significantly prefer manual services to algorithms in the field of medicine [16-18]. Individuals have an aversion to algorithms underlying digital technology, especially when they see errors in the algorithm’s functioning [18]. The preference for algorithms does not increase even if the residents are told that the algorithm outperforms human doctors [19,20]. Our results confirm that fundus image quality in the exposure group is similar to that in the control group in our study, and both are similar to or even better than those reported in previous studies [21,22]. However, the preference for facilitated self-service fundus disease screening is significantly less than that for traditional manual screening. One possible explanation is that uniqueness neglect—a concern that algorithm providers are less able than human providers to account for residents’ (or patients’) unique characteristics and circumstances—drives consumer resistance to digital medical technology [23]. Therefore, personalized health management solutions based on large language models should be developed urgently [24] to meet the residents’ individual demands. In addition, a survey of population preferences for medical AI indicated that the most important factor for the public is that physicians are ultimately responsible for diagnosis and treatment planning [25]. As a result, man-machine collaboration, such as human supervision, is still necessary [26], especially in the early stages of digital transformation to help residents understand and accept the digital technologies.

Furthermore, our study suggests that distrust in the results of facilitated self-service fundus disease screening may cause residents to abandon eye disease screening, irrespective of whether it is provided using this new screening pattern or via the traditional manual screening pattern. This is critical to digital transformation in medicine. This implies that if the digital technology does not perform well, residents will not only be averse to the digital technology itself but also be more likely to abandon health care services as a whole. Digital transformation is a fundamental change to the health care delivery system. This implies that it can self-disrupt its ability to question the practices and production models of existing health care services. As a result, it may become incompatible with the existing models, processes, activities, and even cultures [27]. Therefore, it is important to assess whether the adoption of digital technologies contributes to health system objectives in an optimal manner, and this assessment should be carried out at the level of health services but not at the level of digital transformation [28].

The most prominent limitation of our study is that it was conducted only in Shanghai, China. Because of the sound health care system in Shanghai, residents have already received high-quality eye disease screening services before the adoption of the facilitated self-service eye screening pattern. Consequently, residents are bound to demand more from this new pattern. This situation is quite different from that in lower-income regions. Digital technology was adapted in poverty-stricken areas to build an eye care system, but it did not replace the original system that is based on manually delivered services [13]. Therefore, the framing effect may be weak [29], and there is little practical value in comparing digital technology and manual services in these regions. Second, our study is an observational study and blind grouping was not practical due to the special characteristics of fundus examination. However, we have attempted to use blind processing whenever possible. For instance, ophthalmologists’ evaluation of image quality was conducted in a blinded manner. Third, the manner in which we inquired about residents’ preferences might affect the results. For example, participants in the exposure group generally have experience with manual screening, but those in the control group may not have had enough experience with facilitated screening despite having been shown a video. This might make the participants in the control group more likely to choose manual screening because the new technology was unfamiliar. Finally, individual-level socioeconomic factors or educational level were not recorded, so we cannot rule out the influence of these factors on residents’ preferences.

In summary, this study confirms that the facilitated self-service fundus disease screening pattern could achieve high service quality. The preference of the residents for this new mode, however, was not ideal. It was difficult to reverse residents’ preference for manual screening in a short period, especially when the original manual service was already excellent. Therefore, the digital transformation of health care must proceed with caution. We suggest that attention be paid to the residents’ individual needs. Although more efficient man-machine collaboration is necessary to help the public understand and accept new technologies, personalized health management solutions based on large language models are required.

## Appendix

Multimedia Appendix 1 Video of the non-mydriatic fundus camera Kestrel-3100m with the self-service module.

Multimedia Appendix 2 Questions for screening service quality.

## Abbreviations

AI: artificial intelligence

## Data Availability

Data are available from the corresponding author upon reasonable request.

## References

[1] GBD 2019 BlindnessVision Impairment Collaborators, Vision Loss Expert Group of the Global Burden of Disease Study (2021). Causes of blindness and vision impairment in 2020 and trends over 30 years, and prevalence of avoidable blindness in relation to VISION 2020: the Right to Sight: an analysis for the Global Burden of Disease Study. Lancet Glob Health.

[2] Marques AP, Ramke J, Cairns J, Butt T, Zhang JH, Muirhead D, Jones I, Tong BA, Swenor BK, Faal H, Bourne RR, Frick KD, Burton MJ (2021). Global economic productivity losses from vision impairment and blindness. EClinicalMedicine.

[3] Jan C, Li S, Kang M, Liu L, Li H, Jin L, Qin X, Congdon N, Wang N (2019). Association of visual acuity with educational outcomes: a prospective cohort study. Br J Ophthalmol.

[4] Chai YX, Gan ATL, Fenwick EK, Sui AY, Tan BKJ, Quek DQY, Qian C, Wong TY, Cheng C, Lamoureux EL, Man REK (2023). Relationship between vision impairment and employment. Br J Ophthalmol.

[5] Nayeni M, Dang A, Mao AJ, Malvankar-Mehta MS (2021). Quality of life of low vision patients: a systematic review and meta-analysis. Can J Ophthalmol.

[6] Wang L, Zhu Z, Scheetz J, He M (2021). Visual impairment and ten-year mortality: the Liwan Eye Study. Eye (Lond).

[7] GBD 2019 BlindnessVision Impairment Collaborators, Vision Loss Expert Group of the Global Burden of Disease Study (2021). Trends in prevalence of blindness and distance and near vision impairment over 30 years: an analysis for the Global Burden of Disease Study. Lancet Glob Health.

[8] Cheng C, Wang N, Wong TY, Congdon N, He M, Wang YX, Braithwaite T, Casson RJ, Cicinelli MV, Das A, Flaxman SR, Jonas JB, Keeffe JE, Kempen JH, Leasher J, Limburg H, Naidoo K, Pesudovs K, Resnikoff S, Silvester AJ, Tahhan N, Taylor HR, Bourne RRA, Vision Loss Expert Group of the Global Burden of Disease Study (2020). Prevalence and causes of vision loss in East Asia in 2015: magnitude, temporal trends and projections. Br J Ophthalmol.

[9] Li JO, Liu H, Ting DS, Jeon S, Chan RP, Kim JE, Sim DA, Thomas PB, Lin H, Chen Y, Sakomoto T, Loewenstein A, Lam DS, Pasquale LR, Wong TY, Lam LA, Ting DS (2021). Digital technology, tele-medicine and artificial intelligence in ophthalmology: a global perspective. Prog Retin Eye Res.

[10] Ting DSW, Pasquale LR, Peng L, Campbell JP, Lee AY, Raman R, Tan GSW, Schmetterer L, Keane PA, Wong TY (2019). Artificial intelligence and deep learning in ophthalmology. Br J Ophthalmol.

[11] Xie Y, Nguyen QD, Hamzah H, Lim G, Bellemo V, Gunasekeran DV, Yip MYT, Qi Lee X, Hsu W, Li Lee M, Tan CS, Tym Wong H, Lamoureux EL, Tan GSW, Wong TY, Finkelstein EA, Ting DSW (2020). Artificial intelligence for teleophthalmology-based diabetic retinopathy screening in a national programme: an economic analysis modelling study. Lancet Digit Health.

[12] Tang J, Liang Y, O'Neill C, Kee F, Jiang J, Congdon N (2019). Cost-effectiveness and cost-utility of population-based glaucoma screening in China: a decision-analytic Markov model. Lancet Glob Health.

[13] Xiao X, Xue L, Ye L, Li H, He Y (2021). Health care cost and benefits of artificial intelligence-assisted population-based glaucoma screening for the elderly in remote areas of China: a cost-offset analysis. BMC Public Health.

[14] Generating Evidence for Artificial Intelligence Based Medical Devices: A Framework for Training Validation and Evaluation. World Health Organization.

[15] The Assessment List for Trustworthy Artificial Intelligence.

[16] Dietvorst BJ, Bharti S (2020). People reject algorithms in uncertain decision domains because they have diminishing sensitivity to forecasting error. Psychol Sci.

[17] DeCamp M, Tilburt JC (2019). Why we cannot trust artificial intelligence in medicine. Lancet Digit Health.

[18] Frank D, Elbæk Christian T, Børsting Caroline Kjær, Mitkidis P, Otterbring T, Borau S (2021). Drivers and social implications of artificial intelligence adoption in healthcare during the COVID-19 pandemic. PLoS One.

[19] Juravle G, Boudouraki A, Terziyska M, Rezlescu C (2020). Trust in artificial intelligence for medical diagnoses. Prog Brain Res.

[20] Liu X, Faes L, Kale AU, Wagner SK, Fu DJ, Bruynseels A, Mahendiran T, Moraes G, Shamdas M, Kern C, Ledsam JR, Schmid MK, Balaskas K, Topol EJ, Bachmann LM, Keane PA, Denniston AK (2019). A comparison of deep learning performance against health-care professionals in detecting diseases from medical imaging: a systematic review and meta-analysis. Lancet Digit Health.

[21] Scanlon Peter Henry, Foy Chris, Malhotra Raman, Aldington Stephen J (2005). The influence of age, duration of diabetes, cataract, and pupil size on image quality in digital photographic retinal screening. Diabetes Care.

[22] Cen L, Ji J, Lin J, Ju S, Lin H, Li T, Wang Y, Yang J, Liu Y, Tan S, Tan L, Li D, Wang Y, Zheng D, Xiong Y, Wu H, Jiang J, Wu Z, Huang D, Shi T, Chen B, Yang J, Zhang X, Luo L, Huang C, Zhang G, Huang Y, Ng TK, Chen H, Chen W, Pang CP, Zhang M (2021). Automatic detection of 39 fundus diseases and conditions in retinal photographs using deep neural networks. Nat Commun.

[23] Longoni C, Bonezzi A, Morewedge C (2019). Resistance to medical artificial intelligence. J Consum Res.

[24] Huang AS, Hirabayashi K, Barna L, Parikh D, Pasquale LR (2024). Assessment of a Large Language Model's Responses to Questions and Cases About Glaucoma and Retina Management. JAMA Ophthalmol.

[25] Ploug T, Sundby A, Moeslund TB, Holm S (2021). Population preferences for performance and explainability of artificial intelligence in health care: choice-based conjoint survey. J Med Internet Res.

[26] Young AT, Amara D, Bhattacharya A, Wei ML (2021). Patient and general public attitudes towards clinical artificial intelligence: a mixed methods systematic review. Lancet Digit Health.

[27] Alami H, Gagnon M, Fortin J (2017). Digital health and the challenge of health systems transformation. Mhealth.

[28] Ricciardi W, Pita Barros Pedro, Bourek A, Brouwer Werner, Kelsey Tim, Lehtonen Lasse, Expert Panel on Effective Ways of Investing in Health (EXPH) (2019). How to govern the digital transformation of health services. Eur J Public Health.

[29] Khan WU, Shachak A, Seto E (2022). Understanding decision-making in the adoption of digital health technology: the role of behavioral economics' prospect theory. J Med Internet Res.

