# Supplementary material for: Service Quality and Residents’ Preferences for Facilitated Self-Service Fundus Disease Screening: Cross-Sectional Study
Source: J Med Internet Res. 2024 Apr 17;26:e45545. doi: 10.2196/45545 (PMC11063888; doi:10.2196/45545)
Supplement: Multimedia Appendix 2 [file jmir_v26i1e45545_app2.docx]

**Appendix 2. Questions for screening service quality**

1. Do you feel physiological dis-comfortable during the screening?
2. There is no physiological dis-comfortableness during the screening.
3. There is a little physiological dis-comfortableness during the screening.
4. There is much physiological dis-comfortableness during the screening.
5. Do you feel the screening as safe?
6. The screening is very safe.
7. The screening is safe.
8. It’s hard to say.
9. The screening is unsafe.
10. The screening is very unsafe.
11. Do you feel the screening as convenient?
12. The screening is very convenient.
13. The screening is convenient.
14. It’s hard to say.
15. The screening is inconvenient.
16. The screening is very inconvenient.
17. Do you trust the screening results?
18. The screening result is very trustworthy.
19. The screening result is trustworthy.
20. It’s hard to say.
21. The screening result is untrustworthy.
22. The screening result is very untrustworthy.
